# Association between depression and subjective quality of life among older adults in Japan and Thailand: a cross-cultural multi-group structural equation modeling analysis

**DOI:** 10.1038/s41598-026-51828-w

**Published:** 2026-05-20

**Authors:** Makoto Suzuki, Takako Yamada, Noboru Hasegawa, Tomohiro Umemura, Masahiro Matsunaga, Hunsa Sethabouppha, Nattaya Suwankruhasn, Chalinee Suvanayos, Nobuko Shimizu

**Affiliations:** 1 Faculty of Health Science, Kansai University of Health Sciences, Osaka, Japan; 2 Faculty of Health Science, Bukkyo University, Kyoto, Japan; 3 Graduate School of Nursing, Doshisha Women’s College, Kyoto, Japan; 4 Faculty of Sport Science, Chukyo University, Aichi, Japan; 5 Faculty of Human Health, Aichi Toho University, Aichi, Japan; 6 Faculty of Nursing, Chiang Mai University, Chiang Mai, Thailand; 7 Faculty of Nursing, Toyama Prefectural University, Toyama, Japan

**Keywords:** Quality of life, Depression, Older adults, Structural equation modeling, Cross-cultural comparison, Japan, Thailand, Diseases, Health care, Medical research, Psychology, Psychology

## Abstract

The present study examined the association between depressive symptoms and subjective quality of life (QOL) among older adults in Japan and Thailand. It further analyzed the differences in this structural relationship across cultural contexts. We evaluated the impact of depression on a latent QOL construct and assessed the measurement and structural invariance between the two countries using structural equation modeling (SEM). A cross-sectional survey was conducted among older adults (aged ≥ 60) in urban and rural regions of Japan (n = 88) and Thailand (n = 57). Subjective QOL was assessed using the World Health Organization Quality of Life – Brief Version (WHOQOL-BREF) and modeled as a latent variable comprising five domains. Depressive symptoms were measured using the 15-item Geriatric Depression Scale (GDS-15). SEM with bootstrapping (1,000 resamples) was applied using R and a multi-group analysis was performed to evaluate cross-national differences. Depressive symptoms showed a significant negative effect on latent QOL in the total sample (β = -0.248, p = .008). However, cross-national comparison using chi-square difference test revealed a significant difference in the effect size between Japan (β = -0.49) and Thailand (β = -0.11; Δχ²1= 6.87, p = .008). Thus, measurement invariance was partially supported. Cultural background influences the relationship between depression and QOL. These findings highlight the need for culturally sensitive mental health strategies for older adults.

## Introduction

Considering the global acceleration of population aging, older adults’ quality of life (QOL) has become a critical issue in medical contexts and broader domains such as social policy, community welfare, and mental health^1–3^. QOL is a multidimensional construct that encompasses physical, psychological, social, and environmental aspects. It is a crucial indicator that supports older adults in living with dignity and autonomy^4,5^. Although age-related declines in physical and cognitive functioning negatively impact QOL, emotional factors such as depression also serve as key determinants^6–8^. A growing body of literature acknowledges the significance of enhancing subjective well-being rather than merely preventing disease, highlighting the need for comprehensive support frameworks for improving QOL among older adults^9–11^.

The Geriatric Depression Scale (GDS) has been used in numerous studies demonstrating an association between depression and QOL among older adults^12–14^. Depressive symptoms have been closely associated with poor physical health and reduced social participation, thereby affecting not only psychological well-being but one’s overall QOL as well^6,15–17^. Furthermore, reduced QOL may increase the risk of falls and dementia. Therefore, depression may have implications for both preventative medicine and reduction of caregiving burden^18–20^. However, the strength and direction of these associations may vary depending on cultural, religious, and social contexts^21,22^. Cultural psychology suggests that the association between depressive symptoms and subjective well-being is shaped by culturally embedded emotional norms and coping frameworks. In East Asian contexts such as Japan, emotional internalization may intensify the perceived impact of depressive symptoms on quality of life. In contrast, in Southeast Asian contexts such as Thailand, culturally grounded coping frameworks may influence the interpretation and regulation of emotional distress. These perspectives imply that the strength of the depression–QOL association may vary across cultural contexts. The present study examines this possibility using a multi-group SEM framework comparing Japan and Thailand. In Asian countries, cultural characteristics such as spiritual acceptance and religious values influence caregiving and the psychological burden on caregivers. For instance, the traditional community-based cultural contexts in Thailand have been observed to impose greater emotional and physical burdens on caregivers, highlighting the importance of statistically examining cross-national and cross-cultural differences^23^.

Nevertheless, several previous studies have been conducted in a single country. However, few quantitative studies have compared the structural relationships between depression and QOL across culturally distinct nations^24,25^. Additionally, structural comparisons between countries with significant differences in their cultural, religious, and social welfare systems such as Japan and Thailand are scarce^26^. Moreover, most international studies have relied on mean comparisons or simple regression analyses, with few using structural equation modeling (SEM) to rigorously examine the latent structure of QOL and its influencing factors across countries^27,28^. Thus, it is crucial to explore the effect of cultural backgrounds on the depression-QOL relationship by analyzing measurement and structural invariance.

The present study examined the association between depressive symptoms (GDS-15) and subjective QOL among older adults in Japan and Thailand using SEM. Specifically, QOL was defined as a latent variable constructed from five domains of the World Health Organization Quality of Life – Brief Version (WHOQOL-BREF). Additionally, we evaluated the differences in the effect of depression on QOL by country using multi-group SEM. After analyzing measurement invariance (configural, metric, and scalar), the regression coefficients of the GDS-15 across the two countries were compared using chi-square difference test. Therefore, the objective of this study was to identify whether cultural background influenced the impact of identical depression scores on QOL and to highlight the significance of cultural adaptation in mental health support for older adults.

## Methods

This observational study analyzed cross-sectional quantitative data collected from older adults residing in Japan (Uji City, Kyoto Prefecture; Nagakute City, Aichi Prefecture) and Thailand (Chiang Mai). The study sites in Japan (Uji City in Kyoto Prefecture and Nagakute City in Aichi Prefecture) and Thailand (Chiang Mai) were selected as community-based settings where access to community-dwelling older adults could be facilitated through established local support networks and ongoing research collaborations. These sites provided opportunities to recruit older adults who actively participate in community and health-related activities. However, the sites were selected based on feasibility and accessibility rather than strict national representativeness. Therefore, caution is warranted when generalizing the findings to the broader older adult populations in Japan and Thailand. Standardized questionnaires, including the WHOQOL-BREF and GDS-15, were used to investigate psychosocial and physical characteristics to explore the structural influence of cultural backgrounds on the relationship between depression and QOL. Owing to the non-interventional nature of the present study utilizing secondary data, there was no clinical intervention or random assignment of the participants. SEM was employed to test hypothesized relationships and identify potential cross-national differences in causal structures through assessment of measurement and structural invariance.

Data were collected through self-administered questionnaires distributed to older adults residing in urban and rural areas of Japan and Thailand. Participants in Thailand were recruited through community networks coordinated by the Faculty of Nursing at Chiang Mai University via leaders of local senior groups. In Japan, participants were recruited through local government welfare departments and social welfare councils, which facilitated access to community-based senior groups.

All individuals who attended the health assessment sessions and provided informed consent completed the questionnaire. However, because recruitment was conducted through community-based invitations, the overall response rate could not be precisely determined. In Japan, a small number of individuals (approximately one to two per session) who initially expressed interest did not attend on the day of data collection, while information on non-participants was not systematically recorded in Thailand. The difference in sample size between the two countries reflects differences in recruitment feasibility and participation patterns across settings, including variations in community organization structures and access to eligible participants. Data collection was conducted in 2022 in Japan, whereas data were collected in Thailand in 2023 and 2024. This difference in data collection periods reflects logistical constraints related to the COVID-19 pandemic, including restrictions on international travel and group-based activities in Thailand during earlier periods. The questionnaires were administered using established Japanese and Thai versions of the WHOQOL-BREF and GDS-15, which have been widely used in previous research. Standard translation procedures were followed to ensure conceptual and semantic equivalence across languages. Participants were recruited from local senior centers, community comprehensive support centers, temples, and neighborhood organizations. Written informed consent was obtained from each participant. The Thai sample included communities in rural areas with a strong Buddhist cultural influence, ensuring diversity in the cultural background.

Individuals were eligible to participate if they were aged 60 years or older at the time of the survey and were able to complete all questionnaire items either independently or with assistance. Individuals diagnosed with cognitive impairment or with missing data on key variables were excluded from the analysis.

Subjective QOL was defined as a latent variable (Latent_QOL) based on the five WHOQOL-BREF domains: general life satisfaction, physical health, psychological health, social relationships, and environment. The WHOQOL-BREF is a validated instrument used for cross-cultural QOL assessment^29^. The primary explanatory variable was depressive symptoms, measured using a 15-item GDS. The covariates included age, sex (male = 1, female = 0), physical health measures (30-second chair stand test [CS-30], normal walking speed, and body mass index [BMI]), and social isolation measure (Lubben Social Network Scale-6 [LSNS-6]). Additionally, interaction terms between the country dummy variable and the GDS-15, CS-30, and walking speed were created.

Each WHOQOL-BREF domain score was numerically transformed and used to define the Latent_QOL in the measurement model. All items were assessed for normality and missing data were removed prior to analysis.

All statistical analyses were conducted using R software (version 4.2.3). Bootstrapping (1,000 resamples) was used to estimate standard errors and confidence intervals, providing robustness under limited sample conditions; however, it does not compensate for limited sample size, and the findings should therefore be interpreted as exploratory. The measurement model of Latent_QOL was constructed first, followed by a structural model incorporating the GDS-15 and covariates. Multi-group SEM was used to evaluate measurement invariance across countries (configural, metric, and scalar). Next, cross-national differences in the effect of depression on QOL were assessed using chi-square difference test by comparing a model in which the regression coefficient for the GDS-15 was freely estimated by country versus a constrained model. Additionally, latent scores were extracted, and linear regression analyses were conducted separately for each country to calculate the standardized beta coefficients for the GDS-15. The significance level was set at 5%.

Ethical approvals were obtained from the Central Human Research Ethics Review (CREC030/61BPs, Foundation for Human Research Promotion in Thailand), Aichi Medical University Ethics Review Board (2017-M052, January 30, 2018), and Ethical Review Committee for Research Involving Human Subjects of Toyama Prefectural University (Approval No. KANGO-R4-15, August 31, 2022). All methods were carried out in accordance with relevant guidelines and regulations.

## Results

The final analysis included 145 older adults, including 83 from Japan and 57 from Thailand. Table 1 displays the descriptive statistics for each country. The Japanese participants were older (median age: 75 vs. 68 years), had a higher proportion of males (36.1% vs. 15.8%), showed better physical functioning in the CS-30, and a slightly slower walking speed compared to their Thai counterparts. Data were collected from all participants on five domains: subjective quality of life (QOL), depressive symptoms (GDS-15), physical function (CS-30, walking speed, and BMI), and social isolation (LSNS-6). Eligible participants were aged 60 years or above at the time of the survey and were able to complete all questionnaire items either independently or with assistance. Individuals diagnosed with a cognitive impairment or with missing responses to key variables were excluded from the analysis.

**Table 1:** Sample characteristics by country.

Country	Japan	Thailand
n	83	57
Age (mean)	75.34	68.89
Age (SD)	5.79	6.03
% Male	36.14	15.79
BMI (mean)	23.37	24.62
BMI (SD)	3.37	4.51
CS30 (mean)	19.54	15.24
CS30 (SD)	5.56	5.24
Walk speed (mean)	3.54	3.78
Walk speed (SD)	0.56	0.91
GDS15 (median)	2	3
LSNS-6 (median)	17	16

Note: Values are presented as n (number of participants), mean ± SD, or median. CS30 = 30-second chair stand test; Walk speed = normal walking speed (m/s); GDS15 = Geriatric Depression Scale, 15 items; LSNS-6 = Lubben Social Network Scale, 6 items.

**Fig. 1:**
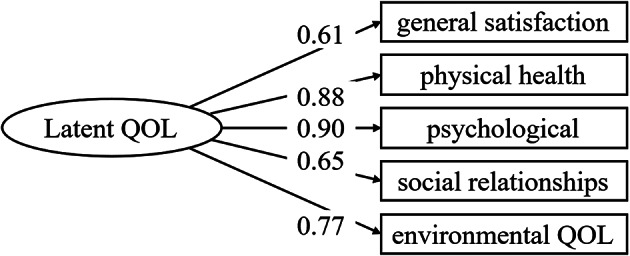
Measurement model of latent QOL using WHOQOL-BREF. *Note*: Standardized factor loadings are shown on the arrows from each observed variable to the latent construct Latent QOL. All loadings were statistically significant at *p* <.001. Latent QOL was constructed from five observed indicators based on the WHOQOL-BREF: general satisfaction, physical health, psychological well-being, social relationships, and environmental quality of life. This measurement model was estimated using structural equation modeling with bootstrap standard errors (1,000 resamples). QOL = Quality of Life; WHOQOL-BREF = World Health Organization Quality of Life – Brief Version.

A measurement model was estimated to validate the latent construct (Latent_QOL) based on the five observed domains of the WHOQOL-BREF: general satisfaction, physical health, psychological well-being, social relationships, and environmental quality of life (Fig. 1). Standardized factor loadings were calculated to analyze the strength of the associations between each observed indicator and the latent variable. The loadings were 0.612, 0.878, 0.898, 0.648, and 0.773 for general satisfaction, physical health, psychological well-being, social relationships, and environmental quality of life, respectively, all of which were statistically significant (*p* <.001). The model showed acceptable fit (CFI = 0.933, TLI = 0.910, RMSEA = 0.069, SRMR = 0.050). Therefore, these results support the unidimensionality and validity of modeling subjective QOL as a single latent construct. Figure 1 presents the measurement model and highlights the observed relationships between the five domains and the latent factor, providing supplementary support for the model’s adequacy.

**Fig. 2:**
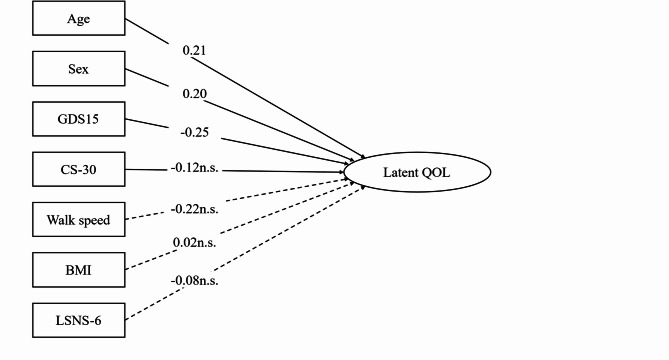
Structural equation model examining associations between depression, covariates, and latent quality of life (Latent QOL). *Note*: Standardized path coefficients (β) are shown. Solid arrows represent statistically significant paths (*p* <.05) and dashed arrows represent non-significant paths (n.s.). Latent QOL = latent variable constructed from the five WHOQOL-BREF domains.

**Fig. 3:**
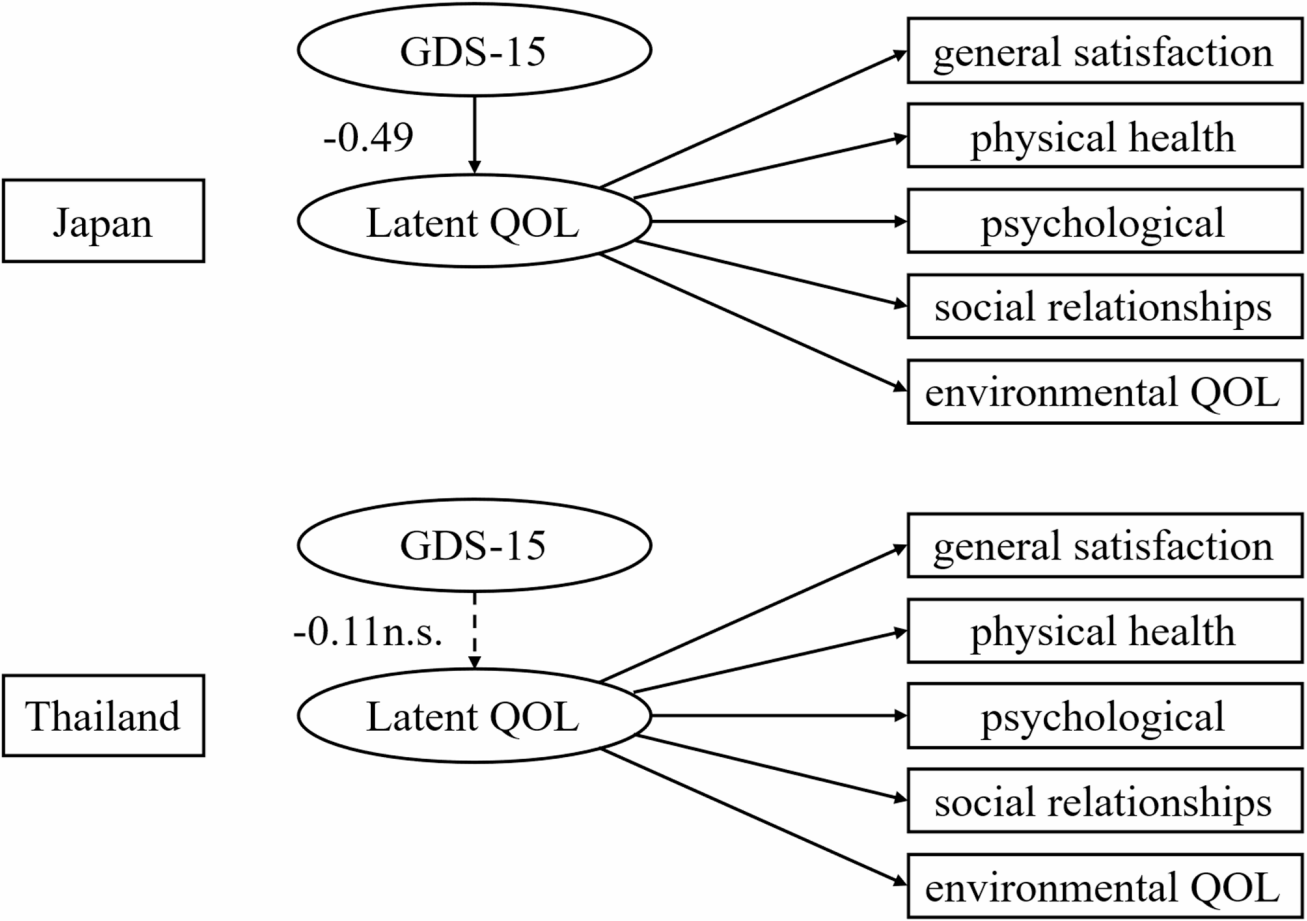
Multi-group structural equation model of QOL among older adults in Japan and Thailand. *Note*: Standardized path coefficients derived from multi-group structural equation modeling are presented for Japanese (left) and Thai (right) older adults. Solid arrows indicate statistically significant paths (*p* <.05) and dashed arrows denote non-significant paths. The latent construct “Latent QOL” is measured by five domains of the WHOQOL-BREF: general satisfaction, physical health, psychological well-being, social relationships, and environmental quality of life. The strength of the association between depressive symptoms (GDS-15) and QOL significantly differed between the two groups (χ² diff^1^= 6.87, *p* =.008).

Next, a structural model was estimated to examine the effects of the GDS-15 and covariates on Latent_QOL (Fig. 2). A significant negative association was observed between GDS-15 and Latent_QOL (β = −0.248, *p* =.008). Age (β = 0.207, *p* =.029) and sex (male = 1, β = 0.204, *p* =.025) were significantly associated with Latent_QOL. In contrast, the CS-30, walking speed, BMI, LSNS-6, and the country dummy variable did not show significant associations. The structural model demonstrated acceptable fit (CFI = 0.933, TLI = 0.910, RMSEA = 0.069, SRMR = 0.050). All estimates were obtained using structural equation modeling with 1,000 bootstrap resamples and statistical significance was determined based on bootstrap confidence intervals. A multi-group SEM was performed separately for the Japanese and Thai participants to determine the cross-national structural differences. As shown in Fig. 3, while Latent_QOL was appropriately measured by five WHOQOL-BREF domains in both countries, the path coefficients from GDS-15 to Latent_QOL differed; a significant negative association was observed in Japan (β = −0.49), whereas the effect in Thailand was small and non-significant (β = −0.11) (Fig. 3).

Configural, metric, and scalar invariance models were tested sequentially to evaluate the measurement invariance across Japan and Thailand. The configural model demonstrated a good fit, supporting equivalence in the latent structure across groups. The metric model, which constrained factor loadings to be equal across countries, showed no substantial degradation in the model fit. However, the scalar model, which further constrained item intercepts, demonstrated a slight decline in the fit indices, suggesting that full scalar invariance was partially supported.

**Fig. 4:**
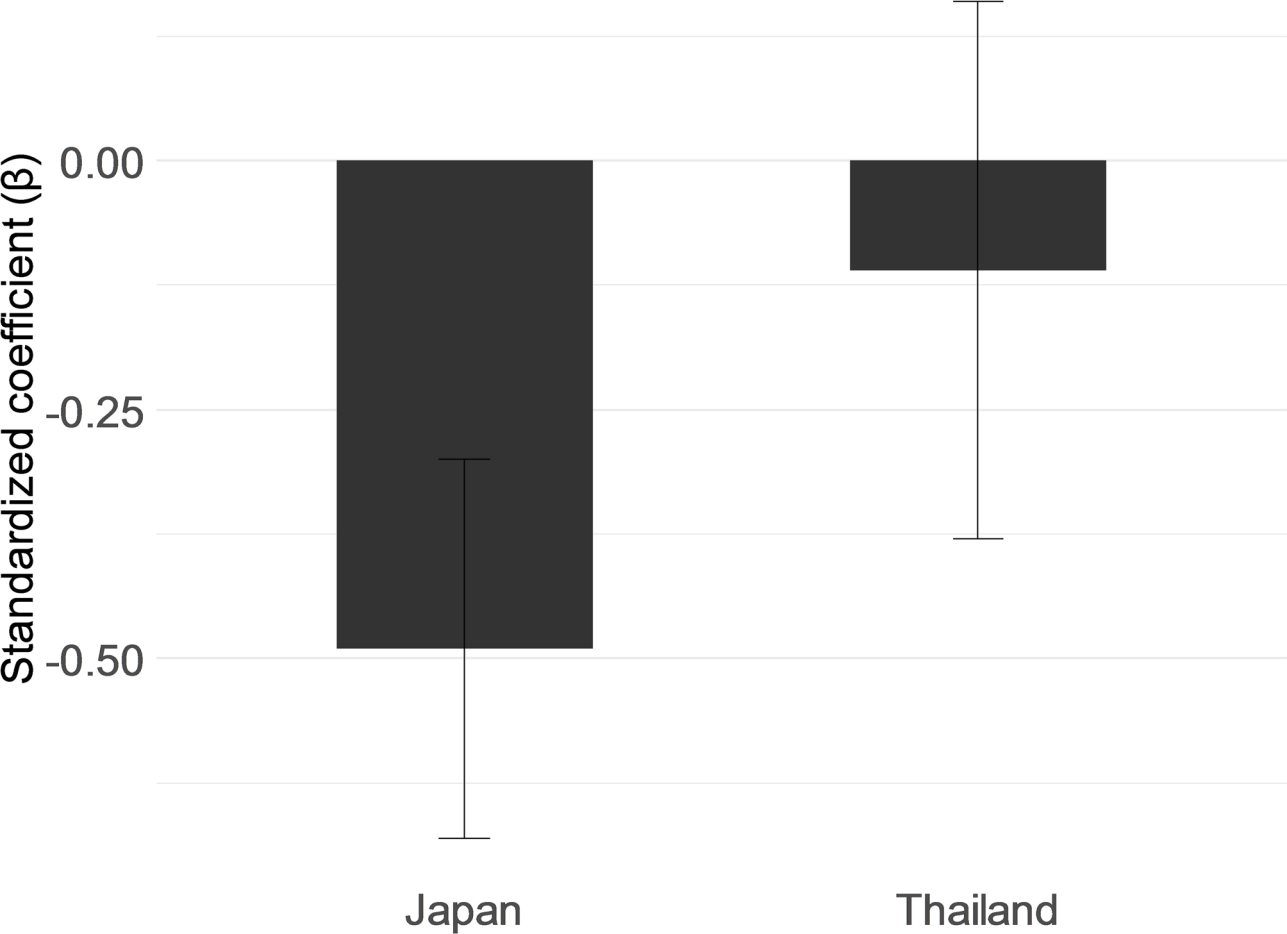
Cross-national difference in regression paths from depression to QOL. *Note*: Bars indicate standardized regression coefficients (β) of GDS-15 predicting latent QOL in each country. Error bars represent 95% confidence intervals.

Two models were compared to examine whether the association between the GDS-15 and Latent_QOL differed between the two countries: a constrained model assuming equal regression coefficients across groups and an unconstrained model allowing coefficients to vary by country. The unconstrained model showed significantly better fit than the constrained model (χ² diff = 6.87, df = 1, *p* =.008), indicating that the effect of the GDS-15 on QOL differed significantly between Japan and Thailand (Fig. 4).

As a supplementary analysis, latent QOL scores were extracted and simple linear regressions were conducted separately for each country using the GDS-15 as a predictor. A moderate negative association was observed among Japanese participants (β = −0.49, 95% CI [−0.68, −0.30]). In contrast, no significant association was found in the Thai group (β = −0.11, 95% CI [−0.38, 0.16]). These findings were consistent with the results of the invariance analysis.

## Discussion

The present study examined whether the association between depressive symptoms and subjective QOL differs between older adults in Japan and Thailand using structural equation modeling. The results showed that depressive symptoms had a significant negative effect on QOL, with a stronger association observed in Japan than in Thailand. The cross-national differences were confirmed using a structural invariance test. In the supplementary regression analyses, a moderate negative association between depression and QOL was found among Japanese older adults, whereas the relationship was weaker and insignificant among Thai older adults. Therefore, the association between depression and QOL may vary across cultural contexts and warrants deeper consideration through a comparison with previous studies.

Through this cross-cultural analysis of older adults in Japan and Thailand, this study provides structural evidence suggesting that the effect of depressive symptoms on subjective QOL may vary according to the cultural background. In Japan, depression had a moderate negative impact on life satisfaction, psychological health, and social relationships, whereas the effect was weaker and statistically insignificant in Thailand. These results suggest that culture-neutral approaches may be insufficient and that mental health support should consider cultural contexts. Future research should employ longitudinal designs to examine the dynamic relationship between depressive symptoms and QOL over time, and incorporate direct measures of culturally relevant factors such as religious practices, coping styles, and social support. In addition, studies using larger and more representative samples are needed to enhance the generalizability and robustness of cross-cultural comparisons.

The study findings are generally consistent with those of prior research on the association between depression and QOL; however, the emphasis on cultural variation offers a novel contribution. In Japan, depressive symptoms assessed using the GDS-15 correlated negatively with physical, psychological, and social aspects of QOL^30^. In particular, domains such as psychological QOL and social relationships are more susceptible to negative emotional states, and depression has been recognized as a key factor in reducing life satisfaction and subjective well-being. In contrast, studies among older adults in Thailand have highlighted the influence of religious values and social acceptance on mental health and subjective happiness, suggesting that clinical definitions of depression (e.g., based on the Diagnostic and Statistical Manual of Mental Disorders [DSM]) may not fully capture their effects on QOL in the Thai context^31^. Thus, the present study contributes methodologically and conceptually by structurally testing these differences and linking cultural psychology with quantitative modeling.

The stronger depression-QOL association observed in Japan may reflect cultural and social influences. In the Japanese society, individuals tend to internalize emotions. The feelings of depression or helplessness may translate more readily into perceived self-devaluation or life dissatisfaction^32^. In contrast, the Thai participants in this study resided in areas where cultural and community-based practices, including those related to religion, may have shaped how emotional distress was perceived and managed. However, because religious beliefs, practices, and coping styles were not directly measured in the present study, this interpretation should be regarded as exploratory^33,34^. Moreover, in Japan, the increasing proportion of older adults living alone and the weakening of community ties may amplify loneliness^35^, thereby intensifying the negative impact of depression on QOL. In Thailand, multigenerational co-residence and temple-centered community structures remain prevalent, enabling social support that may buffer depressive symptoms^36^. These findings point to the need for culturally sensitive approaches to mental health support in cross-cultural research.

The observed cross-national structural differences in the QOL model highlight important considerations for future international and intervention studies. Specifically, the variation in the direction and magnitude of the depression-QOL association suggests that broader cultural contexts, including value systems, family structures, social institutions, and possible differences in meaning and expression, may influence how psychological measures are interpreted across settings. Moreover, since QOL itself is a culturally constructed concept, international comparisons using quantitative instruments must ensure not only measurement invariance, but also culturally informed interpretation and translation frameworks. Future research should adopt mixed-methods approaches that integrate qualitative and quantitative data to explore the cultural meaning of depression, well-being, and QOL^37^.

Methodologically, this study’s use of SEM and invariance testing across countries provides a rigorous basis for detecting structural differences in the depression-QOL relationship. The application of bootstrapping (1,000 resamples) enhanced the robustness of the parameter estimates despite sample size limitations. This approach addresses a critical gap in international studies, which have often utilized mean comparisons or simple regression analyses. By rigorously analyzing both measurement and structural models, this study provides deeper insight into how psychological structures may vary across cultural settings, thereby advancing a culturally informed perspective in QOL research^38,39^.

Moreover, this study reconsiders the cultural validity of assessment tools. Although the GDS-15 and WHOQOL-BREF are used internationally, the interpretation of each item may vary across cultures. For example, the item “I feel that my life has meaning” may carry very different implications in cultures emphasizing self-actualization compared to those rooted in religious or fatalistic worldviews. These context-dependent interpretations highlight the need for a stricter evaluation of measurement invariance in cross-cultural research and serve as important guidance for scale design and translation processes in multinational studies^29,40^.

Therefore, the present findings suggest that cultural background may influence the relationship between mental health and subjective QOL, which is a highly significant insight for public health. One-size-fits-all interventions may be less effective in culturally distinct settings^41,42^. In Japan, individualism and rising social isolation may magnify the effect of depression on QOL, whereas the presence of religious and community networks may serve as psychological buffers in Thailand^31,33,35^. Therefore, the design and implementation of mental health interventions for older adults must be grounded in localized cultural models with strategies tailored to the sociocultural context. For example, in Japan, where depressive symptoms showed a stronger association with QOL, interventions that address psychological distress and social isolation may be particularly important, including community-based mental health programs and opportunities for social participation. In contrast, in Thailand, where the association was weaker, interventions that leverage existing community and culturally embedded support systems, including those related to religious or local networks, may play a complementary role in supporting well-being.

This study has several implications for clinical practice. When evaluating depressive symptoms among older adults, it is essential to assess their potential impact on QOL in culturally sensitive ways^28,29^. Mental health professionals, such as psychologists, occupational therapists, and nurses, can interpret QOL scores more accurately by considering the patient’s cultural background, allowing for more individualized intervention plans. Culturally adaptive care goes beyond mere translation; it requires interdisciplinary collaboration across the health, welfare, religious, and community domains. A future challenge lies in building support systems that incorporate cultural identities while leveraging regional resources.

Furthermore, these findings have implications for community-based elderly care policies. Specifically, the observed variation in the depression-QOL relationship across cultures emphasizes the limitations of uniform policy approaches. In Japan, psychological support must be integrated into community interventions within the framework of a comprehensive community care system to reduce social isolation. In Thailand, culturally appropriate strategies may involve enhancing psychosocial care through religious institutions and local networks. Therefore, future policy designs should prioritize the development of culturally adaptive intervention models based on empirical data. The present findings provide a theoretical foundation for such efforts^1^.

Notably, insights gained from this study provide a culturally nuanced perspective that complements existing global frameworks for elder care, such as those promoted by the WHO^1,2^. Many international intervention programs are based on standardized indicators and assume universality in approach; however, there are cultural differences in the recognition and expression of depression and QOL^40^. Accordingly, international policy frameworks should extend beyond numerical equivalence and incorporate philosophical and semantic reflections on what “QOL” and “depression” mean within each cultural context. These findings provide a multidimensional cultural perspective on QOL in older adults.

Additionally, the study findings extend beyond academic interest and offer practical implications for the global design of policies around aging. Although WHO-led frameworks rely on standardized indicators, subjective health outcomes such as depression and QOL are highly dependent on cultural meanings and modes of expression^1,2,29,40^. Despite identical scores, interpretations may differ across countries and cultures, leading to potential gaps in policy implementation. Therefore, a paradigm shift from culture-invariant to culture-sensitive policymaking is needed. This study contributes to this perspective by linking cultural psychology with public health and providing a basis for more context-sensitive policy design.

This study had several limitations. First, causal inferences cannot be made regarding the effects of depression on QOL due to the cross-sectional nature of the present study. Additionally, it is possible that reduced subjective QOL exacerbates depressive symptoms, indicating a bidirectional relationship. The sample size was relatively small for multi-group SEM, which may limit statistical power and the stability of parameter estimates, particularly in invariance testing. Second, the data were collected in different periods in Japan (2022) and Thailand (2023–2024), which may reflect differences in post-pandemic recovery stages. Although both datasets were obtained during the post-pandemic period, temporal variation may have influenced levels of depressive symptoms and subjective quality of life across countries. Therefore, caution is warranted when interpreting cross-national comparisons. In addition, there were differences in age and sex distributions between the Japanese and Thai samples. Although these variables were included as covariates in the model, residual confounding cannot be ruled out, and these differences may have influenced the observed cross-national associations. Third, the use of non-probabilistic sampling limits the generalizability of the findings. In particular, the Thai sample was skewed toward rural areas with strong religious and community influences, which may limit the generalizability of the findings to urban or more secular populations in Thailand. Furthermore, since psychological factors, such as anxiety and resilience, were not considered, all determinants of QOL were not assessed comprehensively. Finally, this study did not include direct measures of religious beliefs, religious practices, or culturally specific coping factors such as social support. Therefore, interpretations regarding the potential role of these factors in shaping the association between depressive symptoms and QOL remain exploratory. Although standardized versions of the instruments were used, subtle differences in interpretation across cultural and linguistic contexts cannot be fully ruled out.

Furthermore, the measurement model for Latent_QOL (comprising five domains from the WHOQOL-BREF) was assumed to have equal factor loadings across Japan and Thailand. However, cultural differences in how individuals conceptualize and prioritize various QOL domains may cause variations in the underlying latent structure. If the factor loadings were freely estimated for each country, potential differences in the measurement structure might have emerged. Therefore, caution is warranted when interpreting between-group differences at the structural level. Future studies should test more rigorously for measurement invariance, including at the configural, metric, and scalar levels, before drawing cross-cultural conclusions based on structural parameters. Additionally, although participants in both Japan and Thailand were recruited from urban and rural areas, the samples did not fully represent the general older population of each nation. Therefore, caution should be exercised when generalizing cultural and religious tendencies across countries. Cultural elements such as religious values and community structures vary greatly by region, and the current findings should be interpreted as context-specific rather than generalizable. Future studies should incorporate more diverse and representative samples to enable more robust cross-cultural comparisons.

Although the present study utilized a quantitative structural approach based on SEM, such methods may not fully capture the deeper, culturally grounded experiences of depression and subjective QOL. Even when two individuals score similarly on the GDS-15, the underlying emotional background and the meaning of their depressive symptoms may differ considerably across cultures^28,29^. In Japan, depression may be more closely associated with feelings of personal responsibility and future insecurity, whereas in Thailand, depression may be associated with changes in familial roles or conflicts with religious obligations. Understanding these differences requires qualitative methods such as narrative analysis and the exploration of inner experiences. Thus, future studies should combine qualitative investigations with SEM-based causal analysis to construct new interdisciplinary frameworks bridging cultural psychology and gerontology, thereby supporting the adoption of mixed methods approaches^37^.

Although SEM is a powerful tool for modeling complex psychological relationships, quantitative analyses have inherent limitations. Irrespective of standardization, tools like the GDS-15 and WHOQOL-BREF may capture only superficial commonalities across cultures and overlook culturally embedded meanings and expressions^29,40^. For instance, older Japanese adults who report dissatisfaction with life may respond to unmet social expectations or the loss of social roles, whereas older Thai adults may respond to familial disharmony or religious disillusionment. Addressing such contextualized meanings requires qualitative techniques such as interviews and participant observation, reinforcing the need for mixed-methods cultural comparison studies^37^.

In summary, this study demonstrated that the association between depressive symptoms and subjective quality of life differs between older adults in Japan and Thailand. The findings suggest that the strength of this relationship may vary across cultural contexts, highlighting the importance of considering contextual factors when interpreting QOL outcomes. These results underscore the relevance of incorporating cultural perspectives into mental health research and practice for older adults.

## Data Availability

The datasets generated and/or analysed during the current study are available from the corresponding author on reasonable request.
